# Supplementary material for: Analysis of White and Dark without Pressure in a Young Myopic Group Based on Ultra-Wide Swept-Source Optical Coherence Tomography Angiography
Source: J Clin Med. 2022 Aug 18;11(16):4830. doi: 10.3390/jcm11164830 (PMC9410463; doi:10.3390/jcm11164830)
Supplement: Supplementary file 1 [file jcm-11-04830-s001.zip › jcm-1819366-supplementary.pdf]

Supplementary Table S1. Baseline data of 50 paired patients with DWOP lesions for SS-OCTA quantitative analysis

Patient number	Age (years)	Gender	Eye with DWOP lesions	Position of DWOP lesions	AL of the DWOP lesions eye (mm)	AL of the other healthy control eye (mm)
N=50	23.48±1.95	F:M=28:22	OD:OS=29:21	MC:NS:NI:TS:TI= 11:18:24:13:22	26.58±0.82	26.56±0.85
01	23	F	OS	NS, NI	25.59	25.39
02	22	F	OD	NI	28.01	27.76
03	25	F	OD	TS, TI	27.18	25.88
04	24	F	OD	NI	25.65	25.55
05	23	M	OS	NS	27.39	26.93
06	23	F	OD	NI	25.68	26.11
07	23	F	OD	MC, NI, TI	26.30	26.07
08	25	M	OD	TS, TI	28.09	28.55
09	21	M	OD	TI	26.96	27.14
10	24	F	OD	TS, TI	27.20	27.13
11	27	M	OS	NS, NI	27.90	27.97
12	26	F	OD	MC	26.81	25.61
13	24	M	OD	NI	26.06	26.02
14	19	F	OD	MC	26.22	25.91
15	22	F	OS	TS, TI	26.24	26.74
16	21	M	OD	TI	26.44	25.36
17	20	M	OD	TS	27.04	26.54
18	20	F	OS	TI	27.49	27.61
19	22	F	OD	NI	25.35	25.40
20	22	F	OD	MC, NI, TI	24.59	24.38
21	25	F	OD	NS	26.20	26.19
22	25	F	OS	NS, NI	27.93	27.63
23	20	F	OD	NS, NI, TS, TI	26.26	26.43
24	21	F	OS	TS, TI	26.94	26.70
25	21	F	OS	NS, NI	26.62	27.07
26	24	F	OD	MC, NS, TI	27.04	27.25
27	22	F	OD	TS, TI	26.34	26.75
28	23	F	OS	MC, NS, NI	27.78	27.71
29	21	F	OS	TI	27.41	27.36
30	23	F	OS	NI	26.97	26.37
31	20	F	OD	NI, TI	26.25	26.01
32	22	F	OS	TI	25.49	25.56
33	22	M	OS	NS, NI	26.75	26.64
34	26	F	OD	NS, NI	25.53	25.56
35	21	F	OD	MC, NI, TI	25.96	26.70
36	28	F	OD	TI	26.33	26.57
37	24	F	OS	TI	25.97	25.78
38	24	F	OD	MC, NI, TI	26.69	26.32

39	23	M	OS	NS, NI	26.11	25.64
40	26	M	OD	MC	25.97	25.71
41	24	F	OD	NS, NI	25.66	25.64
42	20	M	OS	TS, TI	26.85	26.81
43	24	F	OD	NS	25.86	26.19
44	24	M	OS	NS	27.97	27.79
45	23	F	OD	MC, NS, TS	27.36	27.14
46	23	F	OS	NS, NI, TS	25.63	25.27
47	27	F	OS	NI	26.20	26.25
48	23	F	OS	NS, TS	25.94	26.94
49	26	M	OD	MC, NI, TI	26.43	26.18
50	23	M	OS	TS	28.13	27.74

F, Female；M, Male；OD, Oculus Dexter; OS, Oculus Sinister; DWOP, dark without pressure; MC, Macula-Centered; NS, Nasal Superior; NI, Nasal Inferior; TS, Temporal Superior; TI, Temporal Inferior

Supplementary Table S2. Assessment of reproducibility by intraclass correlation coefficients and coefficients of repeatability

Parameters	MC	TS	NS	TI	NI
**Intraclass correlation coefficient**					
VD					
Inner retina	0.997	0.994	0.915	0.994	0.924
SVC	0.923	0.943	0.996	0.948	0.994
DVC	0.996	0.967	0.976	0.971	0.956
CVI	0.989	0.999	0.997	0.965	0.907
Thickness					
All retina	0.987	0.965	0.971	0.955	0.998
Inner retina	0.956	0.994	0.917	0.991	0.911
Outer retina	0.988	0.986	0.908	0.905	0.981
Choroid	0.999	0.983	0.995	0.958	0.979
**Coefficient of repeatability**					
VD (%)					
Inner retina	1.02	0.69	0.92	0.74	0.81
SVC	0.32	0.24	0.15	0.16	0.41
DVC	0.29	0.11	0.30	0.44	0.12
CVI (%)	1.22	2.31	1.50	1.07	2.81
Thickness (μm)					
All retina	11	15	12	10	19
Inner retina	14	9	10	9	16
Outer retina	6	13	10	9	8
Choroid	15	9	10	11	13

MC, Macula-Centered; NS, Nasal Superior; NI, Nasal Inferior; TS, Temporal Superior; TI, Temporal Inferior; AL, axial length; VD, vessel density; SVC, superficial vascular complex; DVC, deep vascular complex; CVI, choroidal vascularity index

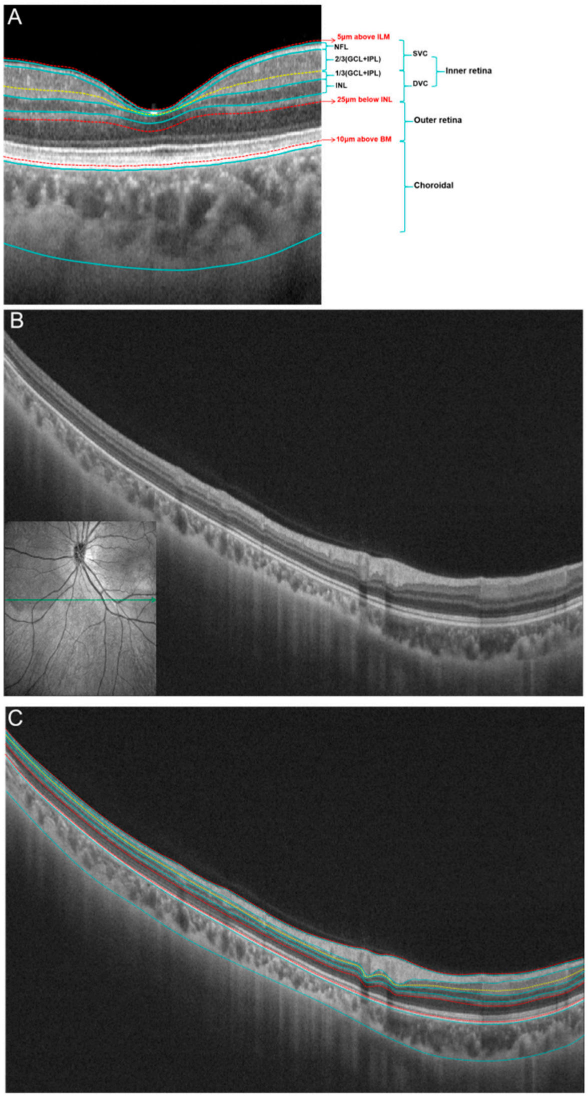

Supplementary Figure S1. One image with stratification details of the peripheral region.

(A) Segmentation of retinal vascular layers and choroidal layers in a 3 mm scan width and 3 mm scan depth OCT B-Scan image centered on the macula. Blue, red, and yellow curves represent segmentation layers. ILM, internal limiting membrane; NFL, nerve fiber layer; GCL, ganglion cell layer; IPL, inner plexiform layer; INL, inner nuclear layer; BM, Bruch's membrane; SVC, superficial vascular complex; DVC, deep vascular complex.

(B) A 12 mm scan width and 3 mm scan depth OCT B-Scan raw image which corresponded to the blue scan line of the 12 mm × 12 mm OCT en-face image (OS, Nasal Inferior) in the lower left corner. The DWOP lesion in the OCT en-face image shows the hyporeflective, and the corresponding OCT B-scan image shows that the ellipsoid zone has faded or even disappeared.

(C) Segmentation of retinal vascular layers and choroidal layers in the OCT B-scan image of **(B)**, in which these stratification lines are the same as in **(A)**.

OCT, optical coherence tomography; OS, Oculus Sinister; DWOP, dark without pressure.
